# The relationships between screen exposure, parent-child interactions and comprehension in 8-month-old infants: The mediating role of shared viewing and parent-child conversation

**DOI:** 10.1371/journal.pone.0296356

**Published:** 2024-01-02

**Authors:** Kexin Tu, Chengwei Shen, Yan Luo, Yushi Mo, Lanying Jian, Xinjie Mei, Qiong Zhang, Lifang Jin, Huiling Qin

**Affiliations:** 1 College of Medical Humanities, Guizhou Medical University, Guiyang, Guizhou, China; 2 Department of Child Health Care, Guiyang Maternal and Child Health Care Hospital, Guiyang, Guizhou, China; 3 College of Public Health, Guizhou Medical University, Guiyang, Guizhou, China; Tallinn University: Tallinna Ulikool, ESTONIA

## Abstract

**Objective:**

To explore the relationships between screen exposure, parent-child interactions and comprehension in 8-month-old infants, and to examine whether shared viewing and parent-child conversation during screen exposure may play mediating role in that relationships.

**Methods:**

The sample included 437 infants aged 8 months from the Children’s Health Department of Guiyang Maternal and Child Health Hospital during January 2022 to February 2023. The use of electronic screen devices was assessed using a screen exposure questionnaire. The Brigance Parent-child interactions Scale was used to assess parent-child interactions and the Putonghua Communicative Development Inventory (PCDI) scale was used to assess infants’ word comprehension.

**Results:**

48.7% of infants were found to be using screens 1–2 days per week. There was a significant difference (p < 0.05) in the PCDI-comprehension scores of screen-exposed infants compared to non-screen-exposed infants. Shared viewing and parent-child conversation during screen exposure were positively associated with parent-child interactions (p < 0.05). Mediation analysis revealed that parent-child conversation fully mediated between screen exposure and PCDI-comprehension, but partially mediated between parent-child interactions and PCDI-comprehension.

**Conclusions:**

Shared viewing and parent-child conversation during screen exposure may mediate between screen exposure and comprehension development. Shared viewing, parent-child conversation and parent-child interactions may be protective factors for screen exposure in comprehension development. Suggests that parents should accompany and communicate with their children when they use electronic screen devices to reduce the negative impact of screen exposure on children’s comprehension.

## Introduction

Word comprehension is the process of acquiring information through perception of words, and it is one of the skills mastered by infants in the earliest stages of language learning. [1, 2]. Recent research indicates that infants begin to comprehend words at 6–9 months [1]. Early word learning can be boosted by comprehension [3], studies have found that parents’ use of spatial language and gestures, as well as home musical environment, can predict infants’ comprehension. [4, 5]. Therefore, exploring other influencing factors on infants’ comprehension may have important implications for children’s language development.

In modern life, electronic devices have become indispensable, with televisions, mobile phones, computers, and other screen media being prevalent in every household. In particular, portable mobile devices like smartphones and tablets have significantly increased the opportunities for infants and young children to interact with screens [6, 7]. Children’s exposure to screens is now increasingly common and continues to trend towards younger ages [8]. In China, children’s exposure to screens has increased dramatically [9].

Children’s exposure to screen has raised concerns for many reasons [10], Studies have shown that screen exposure during early life not only has negative impacts on vision, sleep and weight, but is also negatively correlated with language development [11–15]. However, a recent meta-analysis has found that screen exposure does not have a negative impact on children’s vocabulary, in experimental research, there is actually a positive correlation between screen exposure and vocabulary size [16]. Additionally, there is currently no research examining whether infants’ comprehension is affected by screen exposure. Therefore, in the present study, we examined the following first research hypothesis: (1) Screen exposure may have positive impact on infants’ comprehension directly.

Early childhood is a critical phase for the acquisition of language skills such as phonetics, comprehension and vocabulary [17]. Parent-child interactions are essential in promoting early childhood development, especially in language skills [18–21]. The quantity and quality of parent-child interaction is an important factor in the development of CDI-comprehension and production in children aged 0 to 2 years [22]. Therefore, in the present study, we examined the following second research hypothesis: (2) Parent-child interactions can enhance infants’ comprehension directly.

Children using electronic screen devices on their own may replace opportunities for children and parents to interact and communicate, potentially having indirect negative effects on language development [23]. The recent meta-analysis by Madigan et al. found that shared viewing during screen exposure was associated with improved language skills in children [24]. Moreover, media verbal interactions between mothers and 6-month-old infants during media exposure can moderate the detrimental effects of media exposure on 14-month-old toddlers, and potentially have positive impacts on language development [25].

Therefore, it remains to be further explored whether shared viewing and parent-child conversation during shared viewing (Referencing Shah et al.’s research, the present study defines parent-child conversation during the shared viewing as parent-child conversation [26]) will have an impact on infants’ comprehension development.

In the light of the findings mentioned above, the following third and fourth research hypothesis were examined in this study: (3) Parent-child interactions can affect infants’ comprehension indirectly through shared viewing and parent-child conversation as an intermediary variable. (4) Screen exposure can affect infants’ comprehension indirectly through shared viewing and parent-child conversation as an intermediary variable.

In addition, most of the existing studies were retrospective, and there is limited research specifically focusing on screen exposure of infants. Therefore, this study aims to describe the current status of screen exposure among 8-month-old infants and analyze the relationships between screen exposure, parent-child interactions and comprehension.

## Materials and methods

### Ethical approval

The study was approved by the Institutional Review Board, Guiyang Maternal and Child Health Care Hospital Guiyang Children’s Hospital (No. 2021–65). Guardians were informed of the purpose and procedures of the study and were assured of anonymity. Written informed consent was obtained from all parents or legal guardians.

### Sample

The present analysis included 441 infants from 3 January 2022 to 28 February 2023 who underwent routine physical examinations at the Children’s Health Department of the Guiyang Maternal and Child Health Care Hospital, the refusal rate was less than 3% [27]. Inclusion criteria were intention to receive pediatric primary care in our department for at least 3 years, infants without hearing or visual impairments, and no diagnosed congenital diseases. The general information questionnaire, the screen exposure questionnaire and the Brigance Parent-child interactions Scale were completed online. The Putonghua Communicative Development Inventory scores were obtained through interviews with parents. After excluding irregular and incomplete questionnaires, A total of 437 (238 males and 199 females) participants were retained in the study.

### Research tools

#### General information questionnaire

The general information questionnaire collected basic demographic information about the infants and their parents, including the infant’s birth weight, history of asphyxia, mode of delivery, pregnancy complications, parental education level, family structure and socioeconomic status, as well as family history of mental illness.

#### Screen exposure questionnaire

We prepared a screen exposure questionnaire based on the screen time recommendations by the American Academy of Pediatrics [8] (AAP) and the studies of Wu et al. [28] and Klakk et al. [29]. The questionnaire consisted of four parts, the first part encompassed questions on screen types and contents [30] (Multiple-choice items, parents were asked, "*What types of electronic screen devices has your child been exposed to*?"), the second part encompassed questions on frequency of screen exposure (Parents were asked, "*How many hours a day does your child watch electronic screen devices*?"), the third part encompassed questions on parental intents of screen exposure (Multiple-choice items, parents were asked, "*When would you allow your child to view an electronic screen device*?"*)*, and the fourth part encompassed questions on caregiver’s behavior during viewing (shared viewing and parent-child conversation were determined from this part, parents were asked, "*How often do you watch electronic screen devices with your children when they are exposed to them*?", "*When you and your child watch an electronic screen device together*, *how often do you talk with your child about the screen contents*?" Responses were coded categorically as 1 = never, 5 = always). Cronbach’s alpha for parts 2 and 4 of the questionnaire ranged from 0.896 to 0.912 [31]. Based on the AAP’s guidelines for media usage, no screen time for children under 2, and less than 2 hours per day for children 2 to 12 [32]. The infants in this study were therefore categorized into a screen-exposed group and a non-screen-exposed group [33] (the first item of the questionnaire assessed whether infants had been exposed to electronic screens, and the psychometric properties of the questionnaire were measured by individual items without calculating a composite score).

#### Brigance Parent-child interactions Scale (BPCIS)

The BPCIS developed by Frances Page Glasco was used to test the effect of parent-child interactions [34]. This instrument consists 18 items designed to assess parenting behaviors and perceptions about their children [35]. The questionnaire included item such as ‘‘*I play with my child and show him or her things about toys*.” adapted version of the BPPCIS (5-point Likert scale) was tested for reliability (Internal consistency reliability = 0.794) and validity (Kaiser-Meyer-Olkin value = 0.777 and Bartlett’s test p-value < 0.05) in Chinese families by Jingjing [36]. The Chinese version of the BPCIS adapted by Jingjing was used in the present study to assess the quality of parent-child interactions [36].

#### Putonghua Communicative Development Inventory (PCDI)

The infant language comprehension development was assessed using the PCDI, this is the Chinese version of the MacArthur-Bat Communicative Development Inventory (MCDI) [37]. The PCDI, which consists of infant and toddler forms [38], is a valid tool to assess comprehension and production language skills in infants and young children [39]. In the present study, 8-month-old infants’ word comprehension was assessed by the infant form (Words and Gestures, W&G) [39].

### Quality control

All staff involved in the survey received uniform training, which included instructions on the use of the research instruments and its items. Infant growth and development were assessed by qualified staff with medical training, while other questionnaires were completed by primary caregiver, with guidance and explanation from qualified staff with medical training.

### Statistical analysis

Data was entered in excel and imported into spss version 23.0 (IBM, USA) and jamovi 2.3.12 statistical software for data analysis. The discrete data were described using absolute frequencies and proportions and were analyzed using chi-square tests. Normally distributed continuous data were expressed as mean plus-or-minus standard deviation and independent samples t-test for group comparison; non-normally distributed continuous data were expressed as median (p25, p75) and Mann-Whitney U-test for comparison of two groups, Kruskal-Wallis test for multiple comparisons between groups. Correlations were analyzed using Spearman’s correlation coefficient, Hierarchical multiple regressions and mediating effects tests were used to analyze the factors influencing infants’ comprehension, *p* < 0.05 was considered significant.

## Results

### Descriptive and correlational analyses of the study variables

The 437 infants were divided into screen-exposed group (320, 73.2%) and non-screen-exposed group (117, 26.8%) according to whether they had viewed a screen or not. Smartphones (91.6%) were the most common type of electronic screen device used by infants in the screen-exposed group, with 90 (28.1%) of them having been exposed to a smartphone by 5 months of age. 176 (55%) parents reported that they would allow their child access to screen when using electronic screen device themselves. The differences between the screen-exposed group and non-screen-exposed group were statistically significant in terms of mean monthly income (*χ*^*2*^ = 9.906, *p* = 0.002) and mean time parents spent with their children per day (*χ*^*2*^ = 3.895, *p* = 0.048). The general information of the sample is shown in Table 1.

**Table 1:** The general information of the sample.

	non-screen-exposed group	screen-exposed group		
Gender			0.876	0.024
Male	63 (53.8%)	175 (54.7%)		
Female	54 (46.2%)	145 (55.3%)		
High risk infants			0.369	0.806
No	51 (43.6%)	155(35.9%)		
Yes	66 (56.4%)	165(64.1%)		
Mother’s age	32.9±4.32	32.4±3.62	0.690	0.159
Father’s age	33±4.27	32.3±3.79	0.467	0.529
Mother’s education level			0.203	1.621
Level 1	5 (4.2%)	22 (6.9%)		
Level 2	98 (83.8%)	269 (84.1%)		
Level 3	14 (12%)	29 (9.%)		
Father’s education level			0.059	3.561
Level 1	4 (3.4%)	16 (5%)		
Level 2	96 (82.1%)	277 (86.6%)		
Level 3	17 (14.5%)	27 (8.4%)		
family structure			0.106	2.620
Single parent family	3 (2.6%)	4 (1.3%)		
Joint family	4 (3.4%)	11 (3.4%)		
Immediate family	51 (43.6%)	116 (36.3%)		
Nuclear family	59 (50.4%)	189 (59%)		
Self-reported household income			0.002	9.906
≤4999 RMB/month	1 (0.9%)	19 (6%)		
5000–9999 RMB/month	90 (76.9%)	261 (81.5%)		
≥10000 RMB/month	26 (22.2%)	40 (12.5%)		
parental time with the child(h/day)	4.74±3.71	5.46±4.42	0.048	3.895

Note. Levels of education: 1—high school and below, 2—college and university, 3—postgraduate and above

**Fig 1:**
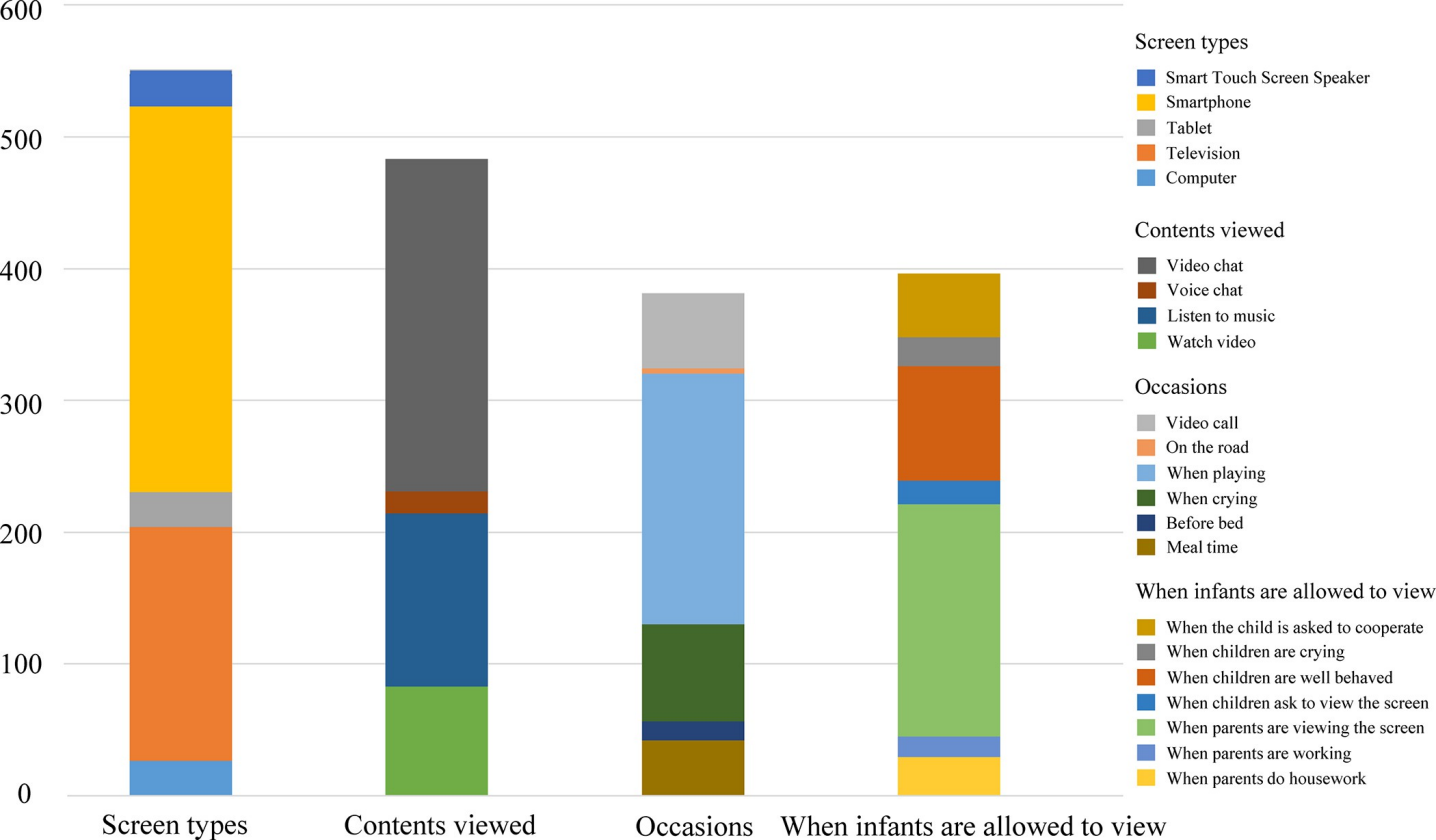
The screen usage of the infants in the screen-exposed group. screen types, contents viewed, occasions and when infants are allowed to view are all multiple-choice items. The Y-axis represents the cumulative number of times that the item has been selected.

289 infants viewed the screen 1–2 times per day (66.1%). 282 infants had screen time of less than 30 minutes per viewing (64.5%). 177 parents did not watch screens with their children, while 240 parents watched screens with their children and talked about the screen contents. The screen usage of the infants in the screen-exposed group is presented in Fig 1. On PCDI-comprehension, there were significant differences in whether infants viewed screens (*U* = 15343.000, *p* = 0.004), screen time per viewing (*χ*^*2*^ = 9.572, *p* = 0.023), frequency of screen exposure per day (*χ*^*2*^ = 9.635, *p* = 0.022), the number of days of screen exposure per week(*χ*^*2*^ = 11.112, *p* = 0.011), shared viewing (*χ*^*2*^ = 14.617, *p* = 0.012) and parent-child conversation (*χ*^*2*^ = 21.936, *p* < 0.001) (Table 2). On the parent-child interactions scores, there were significant differences in screen time per viewing (*χ*^*2*^ = 8.792, *p* = 0.032) and parent-child conversation (*χ*^*2*^ = 22.672, *p* < 0.001) (Table 2).

**Table 2:** Differences between parent-child interactions and PCDI comprehension in screen-exposed infants aged 8 months.

Variable	Cases (n/%)	Parent-child interactions	*χ* ^*2*^ */U*	PCDI-comprehension	*χ* ^*2*^ */U*
screen exposure			18345.000a		15343.000a**
No	117(26.8%)	73(66,79)		34(12,46)	
Yes	320(73.2%)	73.5(68,79)		39(19,58)	
days/week			0.250b		11.112b*
No use	117(26.8%)	73(66,79)		34(12,46)	
≤2days	213(48.7%)	73(68,79)		39(21,59)	
3-5days	81(18.5%)	74(68,80)		39(23,60)	
≥6days	26(6%)	72(67,78.8)		26.5(14,52.3)	
times/day			1.974b		9.635b*
no exposure	117(26.8%)	73(66,79)		34(12,46)	
1-2times	289(66.1%)	73(68,79)		39(18.3,58)	
3-4imes	24(5.5%)	76(70,82.8)		42(29.3,62.5)	
≥5times	7(1.6%)	64(62.5,66.5)		39(30.8,44.3)	
h/time			8.792b*		9.572b*
no exposure	117(26.8%)	73(66,79)		34(12,46)	
<0.5h	282(64.5%)	73(68,79)		39(18.3,58)	
0.5-1h	34(7.8%)	76(70,82.8)		42(29.3,62.5)	
>1h	4(0.9%)	64(62.5,66.5)		39(30.8,44.3)	
shared viewing			5.214b		14.617b**
no exposure	117(26.8%)	73(66,79)		34(12,46)	
never	60(13.7%)	74.5(68.8,77.5)		39(24.8,45.3)	
occasionally	141(32.3%)	74(68,79)		39(19,58)	
sometimes	83(19%)	71(65.5,79.5)		39(14,61)	
often	24(5.5%)	74(68.5,78.3)		55.5(34.8,85.8)	
always	12(2.7%)	78.5(74,83.5)		35(19.3,76.3)	
parent-child conversation			22.672b***		21.936b***
no exposure	117(26.8%)	73(66,79)		34(12,46)	
never	80(18.3%)	72(65.8,77)		39(25.5,44.8)	
occasionally	94(21.5%)	74(68,77.8)		38.5(16,56.5)	
sometimes	91(20.8%)	71(65,79)		39(14.5,63)	
often	31(7.1%)	76(74,79)		63(39.5,85)	
always	24(5.5%)	80(75,83)		39(19.3,58.5)	

Note. a: Mann-Whitney U test, b: Kruskal-Wallis test

*P<0.05

**P<0.01

***P<0.001

Correlation analysis showed that screen exposure was not significantly correlated with parent-child interactions (*r* = 0.015, *p* = 0.749), but screen exposure was significantly correlated with PCDI-comprehension (*r* = 0.139, *p* = 0.004). Parent-child interactions was significantly correlated with PCDI-comprehension (*r* = 0.142, *p* = 0.003) and parent-child conversation (*r* = 0.108, *p* = 0.023) (Table 3).

**Table 3:** The Correlation analysis of screen exposure, parent-child interactions, and l PCDI comprehension in 8-month-old infants.

Variable	1	2	3	4	5	6	7	8	9	10
1.parent-child interaction	—									
2.PCDI-comprehension	0.142**	—								
3. interaction time (h/day)	0.176***	0.106*	—							
4.screen exposure	0.015	0.139**	0.095*	—						
5.day/week	0.013	0.101*	0.080	0.828***	—					
6.time/day	-0.009	0.108*	0.066	0.922***	0.847***	—				
7.h/time	0.041	0.143**	0.039	0.909***	0.838***	0.861***	—			
8.shared viewing	0.006	0.161***	0.111*	0.792***	0.744***	0.778***	0.773***	—		
9.parent-child conversation	0.108*	0.183***	0.126**	0.785***	0.725***	0.755***	0.771***	0.838***	—	
10.video chat time	0.026	0.104*	0.080	0.837***	0.762***	0.807***	0.821***	0.696***	0.696***	—

Note

*P<0.05

** P<0.01

***P<0.001

### Regression and mediating effects analysis

Hierarchical multiple regression was adopted to investigate the impact that socio-demographic details, parental time with the child, parent-child interactions, and screen exposure on the PCDI-comprehension [40]. A total of three models had been adopted: model 1 was inclusive of socio-demographic variables (gender, parental education level, household income); model 2 added parental time with the child; and model 3 added parent-child interactions and screen exposure. The multicollinearity analyses showed that there were little interactions between the factors (all variance inflatable factors were less than 2), There were no signs of multicollinearity in any of the three regression models (Table 4).

**Table 4:** Predictors of PCDI- comprehension: Hierarchical multiple regressions.

	Model 1				Model 2				Model 3			
	*β*	*t*	*P*	*VIF*	*β*	*t*	*P*	*VIF*	*β*	*t*	*P*	*VIF*
Gender	.046	.955	.340	1.007	.047	.975	.330	1.007	.052	1.112	.267	1.008
High risk infants	.076	1.588	.113	1.014	.075	1.560	.120	1.014	.079	1.676	.094	1.017
Mother’s education level	.067	1.256	.210	1.243	.074	1.391	.165	1.249	.061	1.150	.251	1.259
Father’s education level	-.023	-.422	.674	1.259	-.017	-.319	.750	1.262	-.020	-.385	.701	1.269
Self-reported household income	-.039	-.773	.440	1.085	-.039	-.791	.429	1.085	-.029	-.587	.558	1.109
parental time with the child(h/day)					.094	1.961	.050	1.013	.065	1.359	.175	1.041
parent-child interactions									.142	2.961	.003	1.044
screen exposure									.113	2.369	.018	1.036
R^2^	.013				.022				.054**			
Adjusted R^2^	.002				.008				.037**			
R^2^ change	.013				.009				.032**			
*F* change	1.164				3.847				7.277**			

Note

*P<0.05

** P<0.01

***P<0.001

The PCDI-comprehension predictors can be found in Table 4, and model 1 indicated that socio-demographic variables could only explain 1.3% of the PCDI-comprehension variance (*p* > 0.05). After parental time with the child was added to model 2, its overall explanatory power had been increased to 2.2% (*p* > 0.05), parental time with the child had a borderline significant impact on PCDI- comprehension (*β* = 0.094, *p* = 0.05). Model 3 had again added parent-child interactions and screen exposure for the predictive variable. The explanatory power of this model was 5.4%, with an increase of 3.2%, thereby showing that parent-child interactions (*β* = 0.142, *p* = 0.003) and screen exposure (*β* = 0.113, *p* = 0.018) could significantly explain PCDI-comprehension (Table 4).

**Fig 2:**
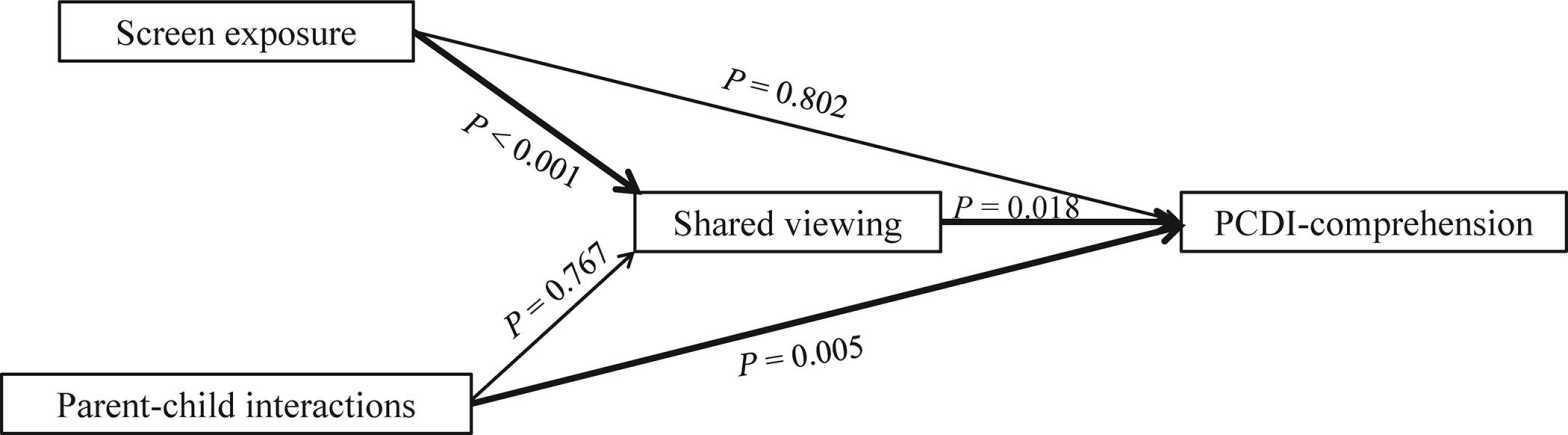
Observed relationships between screen exposure, parent-child interactions, shared viewing and PCDI-comprehension.

**Fig 3:**
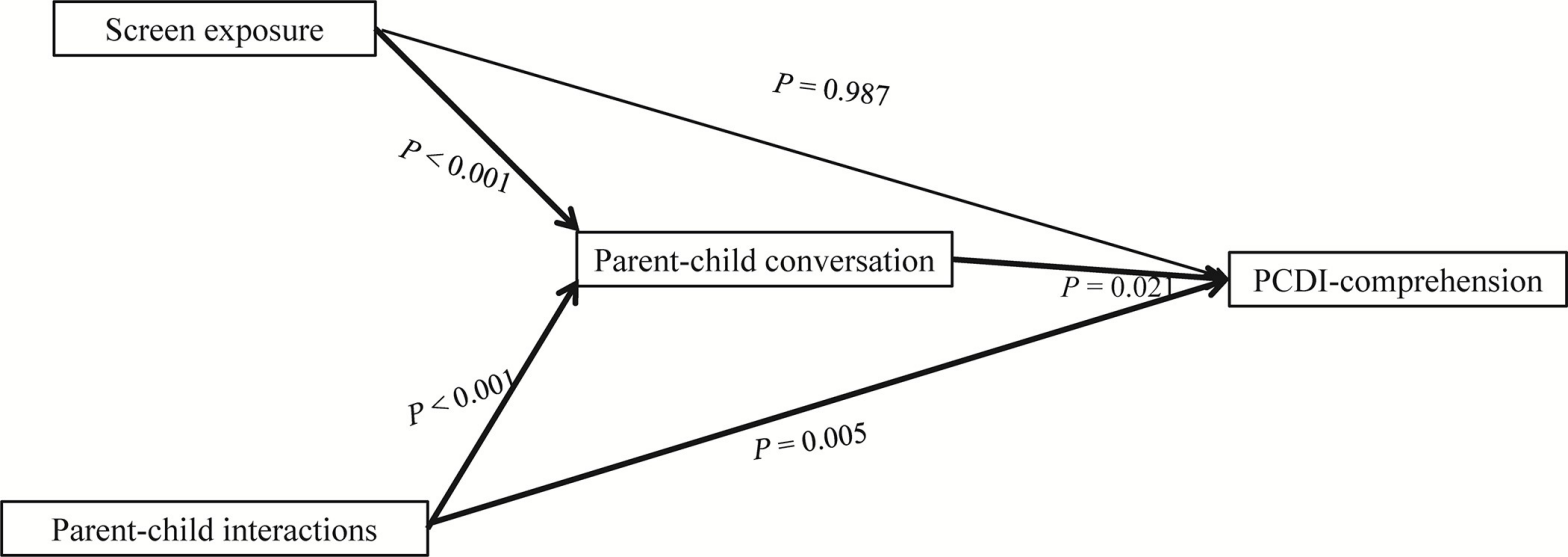
Observed relationships between screen exposure, parent-child interactions, parent-child conversation and PCDI-comprehension.

Through mediating effects analysis, we found that shared viewing (*β* = 0.135, 95% *CI* = 1.420–15.299, *p*_m_ = 0.018) was a full mediator in the relationship between screen exposure and PCDI-comprehension (Table 5 and Fig 2), and parent-child conversation was a full mediator in the relationship between screen exposure and PCDI-comprehension (*β* = 0.118, 95%*CI* = 1.087–13.487, *p*_m_ = 0.021) and parent-child conversation was a partial mediator in the relationship between parent-child interactions and PCDI- comprehension (*β* = 0.020, 95%*CI* = 15.350–0.124, *p*_m_ = 0.047) (Table 6 and Fig 3).

**Table 5:** Results of the mediation analyses of screen exposure, parent-child interactions, shared viewing and PCDI-comprehension.

	effect	SE	β	Z	P	OR(95%CI)
indirect	screen exposure ⇒ PCDI-comprehension	3.541	0.135	2.361	0.018	1.420~15.299
	parent-child interactions ⇒ shared viewing ⇒ PCDI-comprehension	0.017	0.002	0.294	0.769	-0.028~0.038
component	screen exposure ⇒ shared viewing	0.091	0.775	25.605	< .001	2.156~2.513
	screen exposure⇒ PCDI-comprehension	1.510	0.175	2.371	0.018	0.621~6.541
	parent-child interactions ⇒ shared viewing	0.005	0.009	0.296	0.767	-0.008~0.011
direct	screen exposure ⇒ PCDI-comprehension	4.551	-0.019	-0.251	0.802	-10.063~7.777
	parent-child interactions ⇒ PCDI-comprehension	0.147	0.152	3.257	0.001	0.191~0.766
total	screen exposure ⇒ PCDI-comprehension	2.900	0.117	2.489	0.013	1.533~12.901
	parent-child interactions ⇒ PCDI-comprehension	0.148	0.153	3.267	0.001	0.193~0.774

**Table 6:** Results of the mediation analyses of screen exposure, parent-child interactions, parent-child conversation and PCDI-comprehension.

	effect	*SE*	*β*	*Z*	*P*	*OR(95%CI)*
indirect	screen exposure ⇒ parent-child conversation ⇒ PCDI-comprehension	3.163	0.118	2.304	0.021	1.087~13.487
	parent-child interactions ⇒ parent-child conversation ⇒ PCDI-comprehension	0.032	0.020	1.983	0.047	15.350~0.124
component	screen exposure ⇒ parent-child conversation	0.107	0.732	22.853	< .001	2.241~2.662
	parent-child conversation ⇒ PCDI-comprehension	1.284	0.161	2.315	0.021	0.456~5.489
	parent-child interactions ⇒ parent-child conversation	0.005	0.123	3.838	< .001	0.010~0.032
direct	screen exposure ⇒ PCDI-comprehension	4.266	-0.001	-0.017	0.987	-8.431~8.290
	parent-child interactions ⇒ PCDI-comprehension	0.149	0.134	2.818	0.005	0.128~0.714
total	screen exposure ⇒ PCDI-comprehension	2.900	0.117	2.489	0.013	1.533~12.901
	parent-child interactions ⇒ PCDI-comprehension	0.148	0.153	3.267	0.001	0.193~0.774

## Discussion

The present study found that 73.2% of 8-month-old infants had been exposed to electronic screen devices, 55% had been exposed to electronic screens before the age of 5 months, and that smartphones and televisions were the most common types of electronic screen devices that infants were exposed to. 65% of infants used electronic screens 1–2 times per day, with an average of less than half an hour per viewing, the most common use of electronic screen devices by infants was for video chatting with other family members and relatives, shared viewing and parent-child conversation mediated the relationship between screen exposure and PCDI-comprehension.

Previous research has found that the majority of households with children aged 6 months to 4 years have mobile or fixed screens, including televisions (97%), tablets (83%) and smartphones (77%), furthermore, 96.6% of children start using electronic screens before the age of 1 [41]. Zimmerman et al. showed a significant negative correlation between time spent watching videos and PCDI scores for children aged 8 months to 16 months [42]. Shi et al. showed that children with delayed language development were first exposed to screens at 11.6 months of age and spent an average of 118.8 minutes per day on screens, younger age of first screen exposure and longer average daily screen time were associated with reduced parent-child communication and lower levels of language development in children [43]. However, research of highly educated parents found no negative effects of screen exposure on children’s vocabulary, and Taylor et al. suggested that moderate media use will not adversely affect children’s vocabulary development as long as family reading time is not reduced by screen exposure [44].

Recently, a growing body of research suggests that exposure to electronic screens has no negative impact on children’s vocabulary and may even promote early vocabulary development [16, 24]. This may be related to the media contents and the parent-child interactions during screen time [33, 45]. Linebarger et al.’s study found that children’s vocabulary growth was related to the types of screen content [46], and that the infants in the present study were exposed to relatively benign digital media (consisting mainly of video/voice chat and listening to music). Parent-child interactions during screen exposure may be a new form of early childhood education, and such interactions may moderate the impacts of screen exposure by reducing adverse effects and increasing the possibility of benefits [47]. When children are exposed to electronic screen devices, parents can improve their comprehension by co-viewing and engaging in conversation about the contents on the screen. However, this may require benign screen contents as a prerequisite [24, 39, 48].

Comprehension is an important component of early language development in infants [2]. The mediation effect of shared viewing and parent-child conversation in the relationships between screen exposure, parent-child interactions and PCDI- comprehension in the present study seems to be a new finding. The results of the mediating effects analysis may explain why infants in the screen-exposed group had higher language comprehension than the non-exposed group. As infants have difficulty viewing screens alone, parent-child conversation during shared viewing may potentially increase the duration of parent-child interactions, thereby promoting infants’ comprehension. Shared viewing did not play a mediating role in parent-child interactions and comprehension, but parent-child conversation partially mediated this relationship, parent-child conversation may serve as one of the ways for parent-child interactions during screen exposure. Therefore, in order to enhance infants’ comprehension and provide a language-rich environment during shared screen time, parents need to actively interact with their infants and verbally describe the contents on screens [49].

Children’s exposure to electronic screens has become commonplace [49], and the AAP recommends that children younger than 18 months of age should avoid screen exposure [8]. Although many parents are aware of the recommendations, adherence varies widely in actual parental behavior [32]. Given the large number of children prematurely exposed to electronic screen devices, parent-child conversation during shared viewing may play a protective role in language development of screen-exposed children [25, 26, 33, 44].

## Limitations

There are some limitations in the present study. (1) This is a cross-sectional study, and subjects were 8-month-old infants, further prospective cohort studies are needed to explore the causal relationships between screen exposure, parent-child interactions, and language development at different ages. (2) The sample size was small, with only 437 participants. (3) We did not investigate the relationships between screen contents and infants’ comprehension, and future research could explore the long-term effects of screen contents on children’s language development. (4) The questionnaire for screen exposure in the present study was parent-reported. Barr et al. argue that comprehensive and systematic measurement tools of early media exposure are needed [50]. Therefore, future research could use tracking applications on electronic screen devices to objectively monitor screen use, such as the Comprehensive Assessment of Courtroom Media Exposure (CAFE) Consortium [50–52].

## Supporting information

S1 File Raw data. (XLSX)

S2 File Screen exposure questionnaire. (DOCX)

## References

[1] Bergelson E, Swingley D. At 6–9 months, human infants know the meanings of many common nouns. Proc Natl Acad Sci U S A. 2012 Feb 28;109(9):3253–8. doi: 10.1073/pnas.1113380109 Epub 2012 Feb 13. ; PMCID: PMC3295309. 22331874 PMC3295309

[2] Bergelson E. The Comprehension Boost in Early Word Learning: Older Infants Are Better Learners. Child Dev Perspect. 2020 Sep;14(3):142–149. doi: 10.1111/cdep.12373 Epub 2020 Jun 10. ; PMCID: PMC7872330. 33569084 PMC7872330

[3] Syrnyk C, Meints K. Bye-bye mummy—Word comprehension in 9-month-old infants. Br J Dev Psychol. 2017 Jun;35(2):202–217. doi: 10.1111/bjdp.12157 Epub 2016 Sep 13. . 27621053

[4] Kısa YD, Aktan-Erciyes A, Turan E, Göksun T. Parental use of spatial language and gestures in early childhood. Br J Dev Psychol. 2019 Jun;37(2):149–167. doi: 10.1111/bjdp.12263 Epub 2018 Aug 1. . 30069900

[5] Papadimitriou A, Smyth C, Politimou N, Franco F, Stewart L. The impact of the home musical environment on infants’ language development. Infant Behav Dev. 2021 Nov;65:101651. doi: 10.1016/j.infbeh.2021.101651 Epub 2021 Nov 13. . 34784522

[6] Vilela TDR, Rocha MMD, Figlie NB, Mari JJ. Association between psychosocial stressors with emotional and behavioral problems among children of low-income addicted families living in Brazil. Child Abuse Negl. 2019 Jun;92:12–21. doi: 10.1016/j.chiabu.2019.03.005 Epub 2019 Mar 19. . 30901614

[7] Gupta P, Shah D, Bedi N, Galagali P, Dalwai S, Agrawal S, ; et al. J Indian Academy of Pediatrics Guidelines on Screen Time and Digital Wellness in Infants, Children and Adolescents. Indian Pediatr. 2022 Mar 15;59(3):235–244. Epub 2021 Dec 29. . 34969943

[8] Reid Chassiakos YL, Radesky J, Christakis D, Moreno MA, Cross C; COUNCIL ON COMMUNICATIONS AND MEDIA. Children and Adolescents and Digital Media. Pediatrics. 2016 Nov;138(5):e20162593. doi: 10.1542/peds.2016-2593 . 27940795

[9] Dearth-Wesley T, Howard AG, Wang H, Zhang B, Popkin BM. Trends in domain-specific physical activity and sedentary behaviors among Chinese school children, 2004–2011. Int J Behav Nutr Phys Act. 2017 Oct 23;14(1):141. doi: 10.1186/s12966-017-0598-4 ; PMCID: PMC5651590. 29058623 PMC5651590

[10] Huber B, Yeates M, Meyer D, Fleckhammer L, Kaufman J. The effects of screen media content on young children’s executive functioning. J Exp Child Psychol. 2018 Jun;170:72–85. doi: 10.1016/j.jecp.2018.01.006 Epub 2018 Feb 12. . 29448235

[11] Lin YY, Lee WT, Yang HL, Weng WC, Lee CC, Jeng SF, et al. Screen Time Exposure and Altered Sleep in Young Children With Epilepsy. J Nurs Scholarsh. 2020 Jul;52(4):352–359. doi: 10.1111/jnu.12558 Epub 2020 May 12. . 32396281

[12] Krcmar M, Cingel DP. Do Young Children Really Learn Best From the use of Direct Address in Children’s Television? Media Psychol. 2019 Jan 2;22(1):152–171.

[13] Brzozowska I, Sikorska I. Wpływ telewizji na rozwój poznawczy dzieciponiżej 3. roku życia-przegląd badań [Potential effects of screen media on cognitive development among children under 3 years old: review of literature]. Dev Period Med. 2016 Jan-Mar;20(1):75–81. Polish. . 27416629

[14] Huang L, Yang GY, Schmid KL, Chen JY, Li CG, He GH, et al. Screen Exposure during Early Life and the Increased Risk of Astigmatism among Preschool Children: Findings from Longhua Child Cohort Study. Int J Environ Res Public Health. 2020 Mar 26;17(7):2216. doi: 10.3390/ijerph17072216 ; PMCID: PMC7177845. 32224959 PMC7177845

[15] Xu MY, Ren F, Shen LX, et al. Influence of the screen exposure on language development in children under 3 years old. J Clin Pediatr, 2019 Feb; 37(2): 97–101.

[16] Jing M, Ye T, Kirkorian HL, Mares ML. Screen media exposure and young children’s vocabulary learning and development: A meta-analysis. Child Dev. 2023 Apr 12. doi: 10.1111/cdev.13927 Epub ahead of print. . 37042116

[17] Muppalla SK, Vuppalapati S, Reddy Pulliahgaru A, Sreenivasulu H. Effects of Excessive Screen Time on Child Development: An Updated Review and Strategies for Management. Cureus. 2023 Jun 18;15(6):e40608. doi: 10.7759/cureus.40608 ; PMCID: PMC10353947. 37476119 PMC10353947

[18] Glascoe FP, Leew S. Parenting behaviors, perceptions, and psychosocial risk: impacts on young children’s development. Pediatrics. 2010 Feb;125(2):313–9. doi: 10.1542/peds.2008-3129 Epub 2010 Jan 25. . 20100743

[19] Christakis DA, Lowry SJ, Goldberg G, Violette H, Garrison MM. Assessment of a Parent-Child Interaction Intervention for Language Development in Children. JAMA Netw Open. 2019 Jun 5;2(6):e195738. doi: 10.1001/jamanetworkopen.2019.5738 ; PMCID: PMC6575141. 31199447 PMC6575141

[20] Tereno S, Savelon SV, Guedeney A. Preventive parent-young child interaction interventions to promote optimal attachment. Curr Opin Psychiatry. 2019 Nov;32(6):542–548. doi: 10.1097/YCO.0000000000000552 . 31343418

[21] Anderson SE, Keim SA. Parent-Child Interaction, Self-Regulation, and Obesity Prevention in Early Childhood. Curr Obes Rep. 2016 Jun;5(2):192–200. doi: 10.1007/s13679-016-0208-9 ; PMCID: PMC4856567. 27037572 PMC4856567

[22] Robb MB, Richert RA, Wartella EA. Just a talking book? Word learning from watching baby videos. Br J Dev Psychol. 2009 Mar;27(Pt 1):27–45. doi: 10.1348/026151008x320156 . 19972661

[23] Mustonen R, Torppa R, Stolt S. Screen Time of Preschool-Aged Children and Their Mothers, and Children’s Language Development. Children (Basel). 2022 Oct 18;9(10):1577. doi: 10.3390/children9101577 ; PMCID: PMC9601267. 36291513 PMC9601267

[24] Madigan S, McArthur BA, Anhorn C, Eirich R, Christakis DA. Associations Between Screen Use and Child Language Skills: A Systematic Review and Meta-analysis. JAMA Pediatr. 2020 Jul 1;174(7):665–675. doi: 10.1001/jamapediatrics.2020.0327 Erratum in: Pediatr JAMA. 2022 May 1;176(5):528. ; PMCID: PMC7091394. 32202633 PMC7091394

[25] Mendelsohn AL, Brockmeyer CA, Dreyer BP, Fierman AH, Berkule-Silberman SB, Tomopoulos S. Do Verbal Interactions with Infants During Electronic Media Exposure Mitigate Adverse Impacts on their Language Development as Toddlers? Infant Child Dev. 2010 Nov;19(6):577–593. doi: 10.1002/icd.711 ; PMCID: PMC3095495. 21593996 PMC3095495

[26] Shah PE, Hirsh-Pasek K, Kashdan TB, Harrison K, Rosenblum K, Weeks HM, et al. Daily television exposure, parent conversation during shared television viewing and socioeconomic status: Associations with curiosity at kindergarten. PLoS One. 2021 Oct 28;16(10):e0258572. doi: 10.1371/journal.pone.0258572 ; PMCID: PMC8553096. 34710118 PMC8553096

[27] Basu P, Mahajan M, Patira N, Prasad S, Mogri S, Muwonge R, et al. A pilot study to evaluate home-based screening for the common non-communicable diseases by a dedicated cadre of community health workers in a rural setting in India. BMC Public Health. 2019 Jan 3;19(1):14. doi: 10.1186/s12889-018-6350-4 ; PMCID: PMC6318877. 30606132 PMC6318877

[28] Wu XY, Tao SM, Zhang SC, Zhang YK, Huang K, Tao FB. [Analysis on risk factors of screen time among Chinese primary and middle school students in 12 provinces]. Zhonghua Yu Fang Yi Xue Za Zhi. 2016 Jun;50(6):508–13. Chinese. doi: 10.3760/cma.j.issn.0253-9624.2016.06.007 . 27256730

[29] Klakk H, Wester CT, Olesen LG, Rasmussen MG, Kristensen PL, Pedersen J, et al. The development of a questionnaire to assess leisure time screen-based media use and its proximal correlates in children (SCREENS-Q). BMC Public Health. 2020 May 12;20(1):664. doi: 10.1186/s12889-020-08810-6 ; PMCID: PMC7216486. 32397984 PMC7216486

[30] Thomas G, Bennie JA, De Cocker K, Dwi Andriyani F, Booker B, Biddle SJH. Using Wearable Cameras to Categorize the Type and Context of Screen-Based Behaviors Among Adolescents: Observational Study. JMIR Pediatr Parent. 2022 Mar 21;5(1):e28208. doi: 10.2196/28208 ; PMCID: PMC8981006. 35311672 PMC8981006

[31] Lin B, Mei Y, Ma F, Zhang Z, Chen Q, Wang S. Testing the validity and reliability of the Self-Administration of Medication (SAM) instrument in Chinese chronic disease patients: A cross-cultural adaptation. Int J Nurs Pract. 2018 Apr;24(2):e12625. doi: 10.1111/ijn.12625 Epub 2018 Feb 18. . 29457315

[32] Lammers SM, Woods RJ, Brotherson SE, Deal JE, Platt CA. Explaining Adherence to American Academy of Pediatrics Screen Time Recommendations With Caregiver Awareness and Parental Motivation Factors: Mixed Methods Study. JMIR Pediatr Parent. 2022 Apr 5;5(2):e29102. doi: 10.2196/29102 ; PMCID: PMC9019621. 35380541 PMC9019621

[33] Zhou SS, Yan SQ, Cao H et al. Prevalence and the risk factors of television viewing by infants and toddlers in Ma′anshan city. Chin J Child Health Care. 2020 Sep;28(1):61–64. doi: 10.11852/zgetbjzz2019-1087

[34] Glascoe FP. The Brigance Infant and Toddler Screen: standardization and validation. J Dev Behav Pediatr. 2002 Jun;23(3):145–50. doi: 10.1097/00004703-200206000-00003 . 12055496

[35] LaRosa AC, Glascoe FP, Macias MM. Parental depressive symptoms: relationship to child development, parenting, health, and results on parent-reported screening tools. J Pediatr. 2009 Jul;155(1):124–8. doi: 10.1016/j.jpeds.2009.02.028 Epub 2009 Apr 24. . 19394044

[36] Jingjing L. The effects of Socioeconomic status and Parent-infant Interactions on early Lexical Development of 12-to 24-month-old Infants. Master’s thesis, Zhejiang University.2013.

[37] Fenson L, Dale PS, Reznick JS, Bates E, Thal DJ, Pethick SJ. Variability in early communicative development. Monogr Soc Res Child Dev. 1994;59(5):1–173; discussion 174–85. . 7845413

[38] Tardif T, Fletcher P, Liang W, Kaciroti N. Early vocabulary development in Mandarin (Putonghua) and Cantonese. J Child Lang. 2009 Nov;36(5):1115–44. doi: 10.1017/S0305000908009185 Epub 2009 May 13. . 19435545

[39] Lu X, Qin Z. Auditory and language development in Mandarin-speaking children after cochlear implantation. Int J Pediatr Otorhinolaryngol. 2018 Apr;107:183–189. doi: 10.1016/j.ijporl.2018.02.006 Epub 2018 Feb 7. . 29501303

[40] Bai YL, Lai LY, Lee BO, Chang YY, Chiou CP. The impact of depression on fatigue in patients with haemodialysis: a correlational study. J Clin Nurs. 2015 Jul;24(13–14):2014–22. doi: 10.1111/jocn.12804 25827047

[41] Kabali HK, Irigoyen MM, Nunez-Davis R, Budacki JG, Mohanty SH, Leister KP, et al. Exposure and Use of Mobile Media Devices by Young Children. Pediatrics. 2015 Dec;136(6):1044–50. doi: 10.1542/peds.2015-2151 Epub 2015 Nov 2. . 26527548

[42] Zimmerman FJ, Christakis DA, Meltzoff AN. Associations between media viewing and language development in children under age 2 years. J Pediatr. 2007 Oct;151(4):364–8. doi: 10.1016/j.jpeds.2007.04.071 Epub 2007 Aug 7. . 17889070

[43] Shi X, Cai S, Ying WU. Associations between early screen exposure and language delay in children. Jiangxi Med J. 2018 Sep; 53(9):905–908.

[44] Taylor G, Monaghan P, Westermann G. Investigating the association between children’s screen media exposure and vocabulary size in the UK. J Child Media. 2018 Jan 2;12(1):51–65.

[45] Yi YE, Zhou YH, Chen K. Television and DVD/VCD exposure in children younger than three years old in Wenzhou city. Chin Prev Med. 2009 Dec;(12):1060–1064.

[46] Linebarger DL, Walker D. Infants’ and Toddlers’ Television Viewing and Language Outcomes. American Behavioral Scientist. SAGE Publications Inc; 2005 Jan 1;48(5):624–645.

[47] Mendelsohn AL, Berkule SB, Tomopoulos S, Tamis-LeMonda CS, Huberman HS, Alvir J, et al. Infant television and video exposure associated with limited parent-child verbal interactions in low socioeconomic status households. Arch Pediatr Adolesc Med. 2008 May;162(5):411–7. doi: 10.1001/archpedi.162.5.411 ; PMCID: PMC3081686. 18458186 PMC3081686

[48] Guellai B, Somogyi E, Esseily R, Chopin A. Effects of screen exposure on young children’s cognitive development: A review. Front Psychol. 2022 Aug 17;13:923370. doi: 10.3389/fpsyg.2022.923370 ; PMCID: PMC9431368. 36059724 PMC9431368

[49] Wiltshire CA, Troller-Renfree SV, Giebler MA, Noble KG. Associations among average parental educational attainment, maternal stress, and infant screen exposure at 6 months of age. Infant Behav Dev. 2021 Nov;65:101644. doi: 10.1016/j.infbeh.2021.101644 Epub 2021 Sep 9. ; PMCID: PMC8627439. 34509711 PMC8627439

[50] Barr R, Kirkorian H, Radesky J, Coyne S, Nichols D, Blanchfield O, et al. Beyond Screen Time: A Synergistic Approach to a More Comprehensive Assessment of Family Media Exposure During Early Childhood. Front Psychol. 2020 Jul 10;11:1283. doi: 10.3389/fpsyg.2020.01283 ; PMCID: PMC7365934. 32754078 PMC7365934

[51] Madigan S, Browne D, Racine N, Mori C, Tough S. Association Between Screen Time and Children’s Performance on a Developmental Screening Test. JAMA Pediatr. 2019 Mar 1;173(3):244–250. doi: 10.1001/jamapediatrics.2018.5056 30688984 PMC6439882

[52] Radesky JS, Weeks HM, Ball R, Schaller A, Yeo S, Durnez J, et al. Young Children’s Use of Smartphones and Tablets. Pediatrics. 2020 Jul;146(1):e20193518. doi: 10.1542/peds.2019-3518 Epub 2020 Jun 1. ; PMCID: PMC7329252. 32482771 PMC7329252

