# Supplementary material for: The relationships between screen exposure, parent-child interactions and comprehension in 8-month-old infants: The mediating role of shared viewing and parent-child conversation
Source: PLoS One. 2024 Jan 2;19(1):e0296356. doi: 10.1371/journal.pone.0296356 (PMC10760768; doi:10.1371/journal.pone.0296356)
Supplement: S2 File — (DOCX) [file pone.0296356.s002.docx]

**Screen Exposure Questionnaire**

**Exposure to electronic screens:** Children use all types of electronic screens by themselves or with their parents.

**Screen time:** time spent using electronic media such as TV, Internet, computer games, video games, tablets, computers, smartphones, etc.

**1.Is your child exposed to electronic screens?**

a.no(If the parent chooses to answer "NO", the questionnaire will be closed.) b.yes

**2.What types of screens are your children most exposed to at home?(multiple-choice question)**

a. Television. b. computer c. tablet d. smartphone e. smart touch screen speaker

**3. How old was your child when he/she was exposed to the following electronic screens?**

|  | No exposure | <5 months | 5-6 months | 6-7 months | ≥7 months |
| --- | --- | --- | --- | --- | --- |
| Television |  |  |  |  |  |
| computer |  |  |  |  |  |
| tablet |  |  |  |  |  |
| smartphone |  |  |  |  |  |
| smart touch screen speaker |  |  |  |  |  |

**4. How many apps have you downloaded for your child ?**

a.no b.1-2 c. 3-4 d. ≥5

**5.What is the main types of screen content to which your child is exposed? ( multiple-choice question)**

a. watch video b. listen to music c. voice chat d. video chat

**6. What type of videos does your child watch?( multiple-choice question)**

a. advertising b. animation c. music d. education

e. live broadcast f. games g. video chat h. news

**7. Has your child ever video chatted?**

a.no(If the parent choose NO, go to question 9) b.yes

**8. Who does your child mainly video chat with?**

a. father b. mother c. grandparents d. maternal grandparents

**9. How long does your child video chat on average per day?**

a. hardly ever b. <0.5h c. 0.5-1h d. 1-1.5h e. ≥1.5h

**10. How often is your child exposed to electronic screens during the workday?**

|  | No exposure | 1-2 times/day | 3-4 times/day | ≥5 times/day |
| --- | --- | --- | --- | --- |
| Television |  |  |  |  |
| computer |  |  |  |  |
| tablet |  |  |  |  |
| smartphone |  |  |  |  |
| smart touch screen speaker |  |  |  |  |

**11.How often is your child exposed to electronic screens on weekends?**

|  | No exposure | 1-2 times/day | 3-4 times/day | ≥5 times/day |
| --- | --- | --- | --- | --- |
| Television |  |  |  |  |
| computer |  |  |  |  |
| tablet |  |  |  |  |
| smartphone |  |  |  |  |
| smart touch screen speaker |  |  |  |  |

**12. How often does your child use an electronic screen on average per week?**

|  | No exposure | 1-2 times/day | 3-4 times/day | ≥5 times/day |
| --- | --- | --- | --- | --- |
| Television |  |  |  |  |
| computer |  |  |  |  |
| tablet |  |  |  |  |
| smartphone |  |  |  |  |
| smart touch screen speaker |  |  |  |  |

**13.What is the average number of days per week that children spend using electronic screen?**

a. ≤2 days b. 3-5 days. c. ≥6 days

**14. How many times per day on average does your child use electronic screen?**

a. no exposure b. 1-2 times c.3-4 times d. ≥ 5times

**15. How many hours per time on average does your child use electronic screen?**

a. <0.5 h b. 0.5-1 h c. 1-2 h d. 2-3 h e. ≥3 h

**16. How long does your child exposed to electronic screens each weekend?**

a. <0.5 h b. 0.5-1 h c. 1-2 h d. 2-3 h e. ≥3 h

**17. How long does your child exposed to electronic screens each workday?**

a. <0.5 h b. 0.5-1 h c. 1-2 h d. 2-3 h e. ≥3 h

**18. On what occasions has your child view electronic screen?( multiple-choice question)**

a. meal time b. before bed c. when crying d. when playing e. on the road f. video call

**19.what is the time period of your child asked to view electronic screen?(multiple-choice question)**

a. When your child is bored b. When your child is used to using electronic screens

c. irregular d .When your child watching others use electronic screen e.When your child wants to Video Chat

**20.Do you think using screens will help your child?**

a. not helpful b. less helpful. c. moderately helpful d. more helpful e. very helpful

**21.What do you think are the benefits of your child's exposure to electronic screens?(multiple-choice question)**

a. Acquisition of new knowledge b. Acquisition of skills c. Provides entertainment

d. Parents are not disturbed e. Improves parent-child relationship

f. Promotes social interaction and emotional development g. No benefits

**22.What do you think are the disadvantages of children being exposed to electronic screens?(multiple-choice question)**

a. affecst vision and spine b. affects concentration c. affects speech d. affects sleep

e. affects relationships f. affects imagination g. exposure to bad information

**23. when your child are allowed to view electronic screen?(multiple-choice question)**

a. when parents do housework b. when parents are working c. when parents are viewing the screen d. when children ask to view the screen e. when children are crying

f. when children are well behaved g. when the child’s is asked to cooperate

**24. Do you have the following interactions with your child ?**

|  | Never | Hardly ever | Sometimes | Often | Always |
| --- | --- | --- | --- | --- | --- |
| Shared viewing |  |  |  |  |  |
| Parent-child conversation |  |  |  |  |  |

**25. Do you use electronic screens in front of your child unless required for work?**

a. Never b. Hardly ever c. Sometimes d. Often e. Always

**26. Does your child play with electronic toys?**

a. Never b. Hardly ever c. Sometimes d. Often e. Always
