# Supplementary material for: The relationships between screen exposure, parent-child interactions and comprehension in 8-month-old infants: The mediating role of shared viewing and parent-child conversation
Source: PLoS One. 2024 Jan 2;19(1):e0296356. doi: 10.1371/journal.pone.0296356 (PMC10760768; doi:10.1371/journal.pone.0296356)
Supplement: Attachment — Submitted filename: Response to reviewers.docx [file pone.0296356.s003.docx]

Dear Editors and Reviewers:

Thank you for your letter and for the reviewers’ comments concerning our manuscript entitled “The relationships between screen exposure, parent-child interactions and comprehension in 8-month-old infants: The mediating role of shared viewing and parent-child conversation” (ID: PONE-D-23-17586). Those comments are all valuable and very helpful for revising and improving our paper, as well as the important guiding significance to our researches. We have studied comments carefully and have made correction which we hope meet with approval.

SUGGESTIONS FROM EDITOR

1. Please ensure that your manuscript meets PLOS ONE's style requirements, including those for file naming. The PLOS ONE style templates can be found at 
https://journals.plos.org/plosone/s/file?id=wjVg/PLOSOne_formatting_sample_main_body.pdf and 
https://journals.plos.org/plosone/s/file?id=ba62/PLOSOne_formatting_sample_title_authors_affiliations.pdf

2. Please note that funding information should not appear in any section or other areas of your manuscript. We will only publish funding information present in the Funding Statement section of the online submission form. Please remove any funding-related text from the manuscript.

3. We note that the grant information you provided in the ‘Funding Information’ and ‘Financial Disclosure’ sections do not match.

When you resubmit, please ensure that you provide the correct grant numbers for the awards you received for your study in the ‘Funding Information’ section.

4. Thank you for stating the following financial disclosure: 
   "No"

Please state what role the funders took in the study.  If the funders had no role, please state: "The funders had no role in study design, data collection and analysis, decision to publish, or preparation of the manuscript." 
If this statement is not correct you must amend it as needed. 
Please include this amended Role of Funder statement in your cover letter; we will change the online submission form on your behalf.

5. We note that you have stated that you will provide repository information for your data at acceptance. Should your manuscript be accepted for publication, we will hold it until you provide the relevant accession numbers or DOIs necessary to access your data. If you wish to make changes to your Data Availability statement, please describe these changes in your cover letter and we will update your Data Availability statement to reflect the information you provide.

6. Please include your full ethics statement in the ‘Methods’ section of your manuscript file. In your statement, please include the full name of the IRB or ethics committee who approved or waived your study, as well as whether or not you obtained informed written or verbal consent. If consent was waived for your study, please include this information in your statement as well.

Response to editor

1.We have revised the manuscript according to PLOS ONE's style requirements.

2.We have removed the funding-related text from the manuscript.

3.The relevant parts of the funding information have been reviewed and amended.

4.We have stated (in our cover letter) that the funders had no role in study design, data collection and analysis, decision to publish, or preparation of the manuscript.

5. All relevant data are within the manuscript and its Supporting Information files. And we chose the option “Tick here if your circumstances are not covered by the questions above and you need the journal’s help to make your data available.”

6.The full name of the ethics committee was given in the methods section of the manuscript, and informed written consent was obtained from the participants is also stated in the methods.

Reviewer #1: I thank the authors for this manuscript, which investigate an interesting and timely topic in the language development literature.

Unfortunately, I have to recommend that the manuscript be rejected in its current form, because of important methodological flaws.

First, no information is provided about the screen exposure scales. We know nothing about the composition and validity of constructs. How is the Shared Viewing construct defined? What about the Parent Conversation construct?

More details are provided on the Brigance Parent-child interactions Scale (BPCIS), but this scale does not seem to be specifically related to interactions during screen time. Also, not only we don't have information about the scale's validity, but the scale was manipulated by the authors (two items were added, and the scale was changed to 5 points).

I am also concerned with the fact that background variables are not included in the analysis as control variables. The two groups — screen-exposed v non-exposed children — differ significantly in relation to the amount of time that the parent spends with the child, with parents in the screen-exposed group spending more time with their child during an average day. However, it's not really clear to me how this variable relates to the Parent Conversation, Parent-Child Interactions, and Shared Viewing variables. For what I can see, the higher CDI scores may very well be driven by the fact that parents in the screen-exposed groups are spending more time in one-to-one situations with an adult (vs. one-to-many). We may want to factor this out: Holding constant the amount of time that parents spend with their children, what is the effect of spending some of this time looking at a screen?

I can't see the rationale behind the authors' decision to divide the children in two exposure groups, instead of analyzing exposure as a continuous variable. I worry whether this dichotomization may have biased the analyses.

I also wonder whether the authors are aware of this new meta-analysis, based on 11,413 children aged 0-6 years, showing that screen time (including non-shared screen time) has no negative effect on vocabulary size and, if anything, it has a small positive effect? The manuscript should be reframed to have less focus on the negative effects of screen exposure.

While it is true that the authors have made their data available, the variable names have not been translated to English, which makes it effectively impossible to use the data set for international researchers.

Responds to the reviewer’s comments:

1.Thanks to your comments, we have translated and uploaded the screen exposure questionnaire, and for co-viewing and parental conversation are also defined in the introduction and methods, and examples of relevant items are given in the methodology section.

2. The Chinese version of the BPCIS was based on a study by Jingjing Lai, who changed the BPCIS to a 5-point scale and analyzed the reliability (Internal consistency reliability = 0.794) and validity (Kaiser-Meyer-Olkin value = 0.777 and Bartlett's test p-value < 0.05). Glasco mentions that the BPCIS is more closely related to language development in her research (Glascoe FP. The Brigance Infant and Toddler Screen: standardization and validation. J Dev Behav Pediatr. 2002 Jun;23(3):145-50. doi: 10.1097/00004703-200206000-00003.), So we chose the BPCIS as the instrument to measure the level of parent-child interaction in this study.

Glasco mentions that the BPCIS is more closely related to language development in her research. (Glascoe, 2002),

3. Thank you very much for this important comment, Hierarchical multiple regressions were conducted to identify the predictors of PCDI-comprehension and control for the effect of parental time with the child on PCDI- comprehension. In model 3, we discovered that parent-child interaction and screen exposure could significantly explain PCDI-comprehension. To consult for multicollinearity between the factors, multicollinearity analyses has been used in each step. The results shows that the interactions between the factors were little because all VIF in collinear diagnostics were less than 2. there were no signs of multicollinearity in any of the 3 regression models. More results from the hierarchical regression are reported in the results section of the manuscript. Thank you again for your comments.

4. Thanks for your comments, due to the design of the questionnaire, screen exposure was not measured as a continuous variable. But We divided screen exposure into more levels (the screen time per viewing, the number of screen exposure per day, The number of days of screen exposure per week), exploring the relationship between these different levels of screen exposure and PCDI-understanding, and more about this is described in the results and discussion of the manuscript.

5. Thank you very much for the information about this new meta-analysis, which we have carefully read and revised our introduction and discussion to take account of the content of this meta-analysis.

6. Thanks for your comments, it was an oversight on our part, we have translated the file into English and uploaded it.

Reviewer #2:

This a Chinese study reporting on screen exposure in 8 months old infants as reported by over 400 parents. The focus on a very young age group as well as being based on a non-western sample could make it an essential contribution to the field.

There are however a few questions/issues that I believe needs to be explained/addressed before a possible publication.

1. Selection of sample. How many families were approached? As it reads now, not a single family denied participating. If so it needs some explanation. The only attrition mentioned is attributed to incomplete questionnaires (n=4).

2. Ethics. Was the Helsinki convention followed and were the families allowed to withdraw their consent without consequences regarding future care?

3. Method. How long time did the assessment take and was it completed at the clinic? Wad it filled in by the mother, father or conjointly?

4.Quality cntrl. What does “uniform training” entail?

5. Instruments. PCDI. Is there a Chinese validation that has been published? The reference is to a clinical study (25). The same goes for the Brigance scale (23). What was the reason changing the scale from 3- to 5-points?

6. Screen exposure questionnaire. Is it available online or by request? Or is it possible to give an example question? Also: How does it relate to the ongoing debate on how to capture screen time in families with young children (e.g., Barr, R., et al. (2020). Beyond Screen Time: A synergistic approach to a more comprehensive assessment of family media exposure during early childhood, Frontiers in Psychology. 11:1283.)

7. Comprehension score. It is maybe a bit surprising that the children not being exposed to any screens have a lower comprehension score (effect size=?). This find would benefit from one or two more sentences. Could it be that the uneven financial distribution between the groups could be an answer? There is also a near significant effect in the mother’s educational level. Or is it the case that video chat has a positive effect and that this is the effect that the study captures?

8. Content. An improvement of the paper would be to highlight content more. It is almost not mentioned in the background or in the discussion. But content matters! It is obvious from Figure 1 that the screen exposure is relatively benign: The digital media content is almost completely made up of video/voice chat plus listening to music. A rough estimate based on the figure suggests that those aspects make up about 85% of the content. This is not a clear message as the paper is currently written.

9. Limitations: I would not call N = 437 for a small sample but that is of course my subjective opinion. I would prefer “a relatively small sample”.
10. Figures: Figure legends are missing. It is unclear what the Y-axis represents in Fig 1, I have assumed minutes.

Responds to the reviewer’s comments:

1. Thank you for your comments, some parents refused to participate, but we did not count the number of refusals, and as far as we can recall no more than 10 refused to participate in this study (parents receive routine physical examinations in our department, and generally have sufficient time to complete the questionnaire).
2. We obtained ethical approval from the Guiyang Maternal and Child Health Care Hospital and the study adhered to the Principles of the Declaration of Helsinki. The families allowed to withdraw their consent without consequences regarding future care.
3. All questionnaires were completed by either the father or the mother (the one who knew the child better) and took approximately 20 minutes to complete, with 62.93% of the total sample completing them in the clinic and 37.07% completing them online, again under the guidance of trained medical staff.
4. The uniform training includes knowledge of the questionnaire items and their use. We have also added the explanation of "uniform training" to the quality control section.
5. There is a published Chinese version of the validation of the PCDI (Tardif T et al., 2009, doi:10.1017/S0305000908009185), as well as the standardization and validation of BPCIS (Glascoe et al, 2002 doi:10.1097/00004703 -200206000-00003), and we have added these two papers to the references. The reason for changing the BPCIS from a 3-point scale to a 5-point scale is that we refer to the study of Jingjing Lai, who translated the BPCIS into Chinese and changed it to a 5-point scale，and analyzed the reliability(Internal consistency reliability = 0.794) and validity(Kaiser-Meyer-Olkin value = 0.777 and Bartlett's test p-value < 0.05). (Jingjing L，2013)
6. We have translated and uploaded the questionnaire, and provided sample question in the methods section. Thank you for the information you provided about the paper, we also read it carefully and contributed to the discussion.
7. Thank you for your comments. Regarding the comprehension score, we reviewed previous research and found a correlation between it and a number of factors, including the level of parental education and the level of family income that you mentioned. However, in terms of correlations, our results showed significant correlations between comprehension scores and parent-child interactions, co-viewing, and parent-child conversation during co-viewing, which we also explain in the discussion.
8. Thanks for your comments, we have made changes to the relevant parts of the discussion in line with your comments.
9. Thanks a lot for your comments.
10. Thank you very much for your comments, the lack of a figure legends was an oversight in our graphing. The Y-axis in Fig 1 represents the cumulative number of times that the item has been selected.
