# Supplementary material for: The relationships between screen exposure, parent-child interactions and comprehension in 8-month-old infants: The mediating role of shared viewing and parent-child conversation
Source: PLoS One. 2024 Jan 2;19(1):e0296356. doi: 10.1371/journal.pone.0296356 (PMC10760768; doi:10.1371/journal.pone.0296356)
Supplement: Attachment — Submitted filename: Response to reviewers.docx [file pone.0296356.s004.docx]

Dear Editors and Reviewers:

Thank you for your letter and for the reviewers’ comments concerning our manuscript entitled “The relationships between screen exposure, parent-child interactions and comprehension in 8-month-old infants: The mediating role of shared viewing and parent-child conversation” (ID: PONE-D-23-17586). Those comments are all valuable and very helpful for revising and improving our paper, as well as the important guiding significance to our researches. We have studied comments carefully and have made correction which we hope meet with approval.

Reviewer #1:

I thank the authors for revising and resubmitting the manuscript. Unfortunately, many of my methodological issues still stand, and going through the manuscript again has brought some new issues to my attention. Therefore, I believe that the manuscript should go through a substantial revision before being considered for publication. In the following, I will only highlight a couple of major issues in the manuscript.

I am still not sure what "screen exposure", "shared viewing", and "parent-child conversations" are. I assume these are constructs exctracted from the attached questionnaire, but there are too many unanswered questions. Who developed the instrument? Has the instrument been validated? How were the constructs generated — theoretically, based on factor analysis, etc.? How valid are these constructs? What are their psychometric properties? How are the composite scores generated — are they sum scores, mean scores, factor loadings etc.? This is the most important predictor in the paper, and we know virtually nothing about it. All these questions should be answered in the text. Attaching the questionnaire does not help in figuring out how the responses were processed, unfortunately.

On p. 6, the authors say: "The questionnaire covers the following main points: whether or not the infant has been exposed to an electronic screen, types of electronic screen device, content viewed, purpose of screen viewing, the age of children's first screen exposure, frequency of screen exposure, the caregiver’s behavior during viewing, the screen interactivity. Sample items of shared viewing and parent-child conversation". However, only three factors are extracted from the questionnaire. Why were some of the theoretically important constructs combined? Was it a data-driven decision?

Also, I have an issue with screen exposure being coded as a yes-no variable. The questionnaire seems to allow for a much more nuanced (continuous) coding of screen exposure. I appreciate the fact that screen exposure may differ across cultures, but I find it hard to believe that there are many infants that have NO interactions whatsoever with digital media, given how ubiquitous they are.

My other big concern relates to the outcome variable. No summary statistics for the PCDI are provided, but I checked myself and the mean comprehension score is around 40 words (SD = 27), which is comparable to CDI data from other languages. However, nothing seems to predict differences in PCDI scores except for parent-child interactions and screen exposure. There are no effects of gender, risk factors (which is not defined in detail), parental education, or household income. This is very unusual, given that all these variables are known predictors of language development already at such an early age. For instance, gender alone is known to explain around 8%/9% of the variance in vocabulary size; therefore, it is puzzling that a model including both background factors and data on parent-child interactions only accounts for around 5% of the variance. I am not saying that the data is incorrect, but it is hard to interpret a model in which many important predictors of language development do not show any effect. Are there other factors that have not been included in the analyses that may explain the variance that is left unexplained by the included variables?

Once these issues have been dealt with, it will be easier to interpret the results (it is not possible at the moment because there are too many unknowns).

Responds to the reviewer’s comments:

1.Thanks for your comments, we have modified the description of the screen exposure questionnaire in the Methods part. We prepared a screen exposure questionnaire based on the screen time recommendations by the American Academy of Pediatrics and the studies of Wu et al., 2016 and Klakk et al., 2020，The questionnaire consisted of four parts, and since the first and third parts of the questionnaire were multiple choice questions for descriptive statistics (The results are presented in Table 2 and Figure 1), we calculated Cronbach's alpha coefficients only for the parts 2 and 4. Regarding the question about the composite scores of the questionnaire, we did not calculate the composite scores, and the results for shared viewing, parent-child conversation, etc. were derived from a single item in the questionnaire.

2. Thanks for your comments. These three factors were our main focus and the main purpose of this research (the role of co-viewing and parent-child conversation during co-viewing in children's screen exposure). Other results of the questionnaire are reported in Table 2 and Figure 1. The scores for these three factors were derived from individual items rather than composite scores.

3. Thanks for your comments. The results of dividing infant screen exposure into more levels of coding are reported in Table 2. Fewer infants in China are screen-exposed than in developed countries (Kabali et al., 2015; Chen et al., 2019) (about 50% of Chinese infants are exposed to electronic screens. Shan et al., 2023), and with reference to the method of Zhou et al., 2020; Chen et al., 2018 and Sun et al., 2020 who coded screen exposure as a yes/no variable. Based on the AAP guidelines on screen use, which state that electronic screens are not recommended for children under 2 years of age, and that children older than 2 years of age should have less than 2 hours of screen time per day. The children in this study were 8 months old and were therefore divided into a screen-exposed group and a non-screen-exposed group. Our screen exposure questionnaire was not designed to take into account the screen as a backdrop (e.g., whether children watch television, rather than whether television is used as a backdrop when children play), so there are many infants that have NO interactions whatsoever with digital media. Chinese society and culture maintains an attitude that screen exposure hampers early childhood development (LI Min-yi et al., 2022), so some parents try to avoid exposing their children to electronic screens. According to the results of previous studies, the proportion of Chinese infants exposed to screens and the duration of exposure is lower than that of infants in developed countries such as the United States and Singapore (Li et al., 2020 - The Relationships between Screen Use and Health Indicators among Infants, Toddlers, and Preschoolers: A Meta-Analysis and Systematic Review).

4. Thank you very much for this important comments. Since the R^2^ in the hierarchical multiple regression is only 5%，similarly, we considered that there might be other predictors of language development (e.g. a rich language environment at home) that were not included in the model, but the background factors we studied only those components that were included in the model. And a small R^2^-value may blur the contribution of background factors such as gender, parental education, household income, risk factors, etc., to PCDI. And the relatively small sample size is also considered to be a possible reason for the small contribution of other background factors. In searching for relevant studies, we found that the contribution of gender to infants' language development is still under discussion (Oller et al., 2023; Hyde & Linn 1998-Gender Differences in Verbal Ability: A Meta-Analysis. Psychological Bulletin). Some studies have found that gender contributes to language development (Shen & wang, 2018; Cao et al., 2019;), but others have found that gender has no significant effect on language development (Hao et al., 2005; Wang et al., 2019; Oller et al., 2023). A Chinese study found that although parental education has a significant effect on infant language, the relationship between maternal education and infant language development is no longer significant after controlling for parenting knowledge and parenting behaviour (Wang et al., 2019). And Bian et al., 2007 found that children in families with moderate social-economic status had the highest levels of language development. (Possibly influenced by China's social and economic environment, where middle-income groups take longer breaks and spend more time with children, promoting children's language development.)

Reviewer #2:

Thanks for the revised version, the paper is now much clearer and in my view closer to be accepted. However I do have some remaining comments that I believe should be addressed.

1. Attrition. There is still no info in the paper re the attrition which I believe is necessary.

2. Ethics. Now ok.

3. Method. The answer is informative, but I would like some of it to be included in the paper as well, e.g., who completed the q-naire (couldn’t find it in the revised text).

4. Quality cntrl. Now ok

5. Validations. Now mostly ok but the text states “We referred to the Chinese version of the BPCIS, which was validated by Jingjing, to evaluate the quality of parent-child interactions”. A ref to Jingjing is missing (29) here.

6. Screen exposure. In your answer you write that the ref I provided (Barr et al., 2020) contributed to the discussion but I cannot see how and the reference is not added. So what do you mean with “contributed to the discussion”?

7- 10: All issues addressed.

Responds to the reviewer’s comments:

1. Thanks for your comments, we've added information about attrition to the methods section.

2. Thank you for this comment.

3. Thanks to your comments, we have included relevant information such as who completed the questionnaire in the quality control section.

4. Thank you for this comment.

5.Thanks to your comments, we have added a reference to Jingjing.

6. Thank you for the reminder and we apologize for the error we made, we have revised the limitations rather than the discussion based on the Barr et al. study. We have added references to the fourth point of the limitations. Your comments are very helpful.
