# Supplementary material for: The relationships between screen exposure, parent-child interactions and comprehension in 8-month-old infants: The mediating role of shared viewing and parent-child conversation
Source: PLoS One. 2024 Jan 2;19(1):e0296356. doi: 10.1371/journal.pone.0296356 (PMC10760768; doi:10.1371/journal.pone.0296356)
Supplement: Attachment — Submitted filename: Response to reviewers.docx [file pone.0296356.s005.docx]

Dear Editors and Reviewers:

Thank you for your letter and for the reviewers’ comments concerning our manuscript entitled “The relationships between screen exposure, parent-child interactions and comprehension in 8-month-old infants: The mediating role of shared viewing and parent-child conversation” (ID: PONE-D-23-17586). We have studied comments carefully and have made correction which we hope meet with approval. Revised portion are marked in yellow in the paper.

Firstly, we would like to express my sincere gratitude for reviewer #2, the comments and suggestions of reviewer #2 have greatly improved the quality of the manuscript. Furthermore, we would also like to extend my heartfelt thanks for the recognition and positive feedback of reviewer #2 on the manuscript.

Reviewer #1 noted in his comment that many of his questions remained unanswered, possibly because we revised the manuscript in accordance with Reviewer #1's comments without providing specific answers to the related questions. Therefore, we have reviewed reviewer #1's comments from the two previous revisions and provided specific answers in this response.

**Reviewer #1:**

The authors answered some of my questions and have made a relatively good case in #4 about why eg. education and child gender do not predict vocabulary development in their model. However, many of my questions are still unanswered.

Responds to the reviewer’s comments:

Thank you for reviewing our manuscript and providing valuable feedback and comments. Your previous comments and suggestions have been very helpful in our revisions. We have re-evaluated your previous comments and provided specific responses. We greatly appreciate your patient guidance, and your input is crucial to our work.

We are very sorry for our negligence in answering the questions relating to the screen exposure questionnaire. In the methods section of the manuscript, we have described the constructs of "screen exposure," "shared viewing," and "parent-child conversation.", the reliability of the questionnaire, the psychometric properties and the composite scores of the questionnaire. In this response, we have answered those questions according to your comments.

We developed the screen exposure questionnaire based on the screen time recommendations by the American Academy of Pediatrics and the studies of Wu et al. (2016)^[1]^ and Klakk et al. (2020)^[2]^. Regarding the issue of whether this instrument has been validated, we have calculated the Cronbach's alpha coefficient (0.896 - 0.912). The questionnaire consisted of 11 items, 4 of which were analyzed by descriptive statistics (Fig 2). Many of the questionnaires used in previous studies^[3]^^[4]^^[5]^^[6]^^[7]^^[8]^^[9]^^[10]^^[11]^^[12]^^[13]^^[14]^^[15]^ on screen exposure were not validated for reliability, and psychological characteristics were obtained from individual items without calculating composite scores. The screen exposure questionnaire used by Çelen Yoldas T et al. (2021)^[16]^, Shah PE et al. (2021)^[17]^, and Lin Y, et al. (2022)^[18]^ , which were referenced in this study, included items on co-viewing and parent-child conversation during screen exposure.

The item in the screen exposure questionnaire used in the present study regarding whether or not the children had been exposed to screens was informed by the study of Zhou et al.^[19]^ (Has your child been exposed to an electronic screen device?). The "shared viewing" item was informed by the study of Zimmerman FJ et al.^[20]^, in this study, parents were asked "*How often do you watch electronic screen devices with your children when they are exposed to them?*" Similarly, Zimmerman FJ et al. used a **single** item to evaluate this psychometric property. The "parent-child conversations" item was informed by the study of Shah PE et al.^[21]^ In the present study, parents were asked "*When you and your child watch an electronic screen device together, how often do you talk with your child about the screen contents?*" Similarly, Shah PE et al. used a **single** item to evaluate this psychometric property and there were only two items on screen exposure in Shah PE et al. study: *Hours of television viewing* and *Parent conversation during shared television viewing.* As in previous studies^[22]^^[23]^^[24]^^[25]^^[26]^^[27]^^[28]^, the present study evaluated the psychometric properties of the screen exposure questionnaire with individual items, and composite scores were not calculated.

Response to comment: However, only three factors are extracted from the questionnaire. Why were some of the theoretically important constructs combined? Was it a data-driven decision?"

Response: We have carefully reviewed the comments. Firstly, the other factors were analyzed by descriptive statistics (see Table 1 and Figure 1). The three factors you mentioned are the ones we intended to explore during the research design phase of this study. We wanted to explore how parental behaviors (co-viewing and talking about screen contents) during infants' exposure to screens had an impact on children's development, and were therefore not data-driven.

Response to comment: Why was screen exposure coded as a yes-no variable？

Response: We have coded the screen exposure as a yes-no variable according to the American Academy of Pediatrics' screen time guidelines (AAP calls for no screen time at all for children until 18 to 24 months), The subjects in studies of Wu et al.^[29]^ and Tombeau Cost K et al.^[30]^ were similarly younger than 18 months, and they also coded screen exposure as a yes-no variable.

Response to comment: it hard to believe that there are many infants that have NO interactions whatsoever with digital media, given how ubiquitous they are.

Response: The screen exposure questionnaire in the present study was not designed to take into account the screen turned on in the background, Therefore, our findings demonstrated that there were many infants that have NO interactions whatsoever with digital media. In Chinese society and culture, attitudes towards electronic screen devices are relatively negative, especially for children's development^[31]^, Therefore, some Chinese parents try to avoid exposing their children to electronic screen devices. According to previous research, compared to developed countries like the United States and Singapore, Chinese infants and young children have lower rates of screen exposure and spend less time using screens^[32]^. Regarding the proportion of infants and toddlers exposed to screens, our results are similar to findings in previous studies that conducted in China^[33]^^[34]^. With respect to the social and cultural differences between China and other developed countries like the United States and those in the European Union, we therefore again answer this important comment.

Regarding issues related to the BPCIS, we noted in the methods section that we have used the Chinese version of the BPCIS revised by Jingjing Lai. Furthermore, we have revised the methods section of the manuscript based on Jingjing Lai's thesis.

We have tried our best to improve the language in the manuscript and have modified some confusing sentences, making them concise and easy to read. We would greatly appreciate it if you could kindly note any other errors so that we may revise our manuscript accordingly based on your valuable suggestions.

If you have any further questions, please do not hesitate to inform us.

**Reviewer #2:**

All of my questions have been fully answered and I the paper is publishable. I congratulate the authors to a nice paper.

Responds to the reviewer’s comments:

We would like to extend my sincere gratitude once again for your endorsement of our manuscript. Your recognition and support are deeply appreciated.

1. Wu XY, Tao SM, Zhang SC, Zhang YK, Huang K, Tao FB. [Analysis on risk factors of screen time among Chinese primary and middle school students in 12 provinces]. Zhonghua Yu Fang Yi Xue Za Zhi. 2016 Jun;50(6):508-13. Chinese. doi: 10.3760/cma.j.issn.0253-9624.2016.06.007. PMID: 27256730.
2. Klakk H, Wester CT, Olesen LG, Rasmussen MG, Kristensen PL, Pedersen J, Grøntved A. The development of a questionnaire to assess leisure time screen-based media use and its proximal correlates in children (SCREENS-Q). BMC Public Health. 2020 May 12;20(1):664. doi: 10.1186/s12889-020-08810-6. PMID: 32397984; PMCID: PMC7216486.
3. He Hui, Wei Zhuang, Liu Chunyang, Liang Aimin. Survey on Children's Use of Electronic Products in Beijing. Maternal and Child Health Care of China. January. 2016;31(2):351–353. doi:10. 7620 /zgfybj. j. issn. 1001－4411. 2016. 02. 47
4. ZHOU Shan-shan, YAN Shuang-qin, CAO Hui, GAO Guo-peng, CAI Zhi-ling, GU Chun-li, LIU Ting-ting, QIAN Zhi-kan, WANG Hao, LIU Zi-jian, TAO Fang-biao. Prevalence and the risk factors of television viewing by infants and toddlers in Ma′anshan city. Chinese Journal of Child Health Care, 2020, 28(1): 61-64.
5. FANG Hong-ying, TANG Yin-xia, XU Cheng-heng,YANG Fei-fei,ChEN Shang-hui. Survey on exposure to electronic devices of preschool children in Tongling city. Chinese Journal of Child Health Care, 2018, 26(8): 913-915.
6. YEYi, ZHOU Yong hai, CHEN Ke. Television and DVD/VCD exposure in children younger than three years old in Wenzhou city. Chinese preventive medicine , 2009, 10(12):1060–1064.
7. WANG Xuena, DU Wenwen, ZHANG Meng, et al. Investigation on screen time and psychosocial problems of preschoolers during the prevalence of COVID—19. China Journal of Health Psychology, 2021;29(4):564–568.
8. SHAN Ruijie, HAN Jing, QU Keli, YUE Lei, CUI Naixue. Associated factors of screen exposure in infants. Chinese Journal of Child Health Care, 2023, 31(4): 374-378.
9. Zhao Shanshan, Yao Conghua, Xu Jinping. Prospective cohort study and risk factor analysis of language delay based on outpatient in Xiamen.Chin J Appl Clin Pediatr, 2021,36(14):1094-1097.
10. Tombeau Cost K, Korczak D, Charach A, Birken C, Maguire JL, Parkin PC, Szatmari P. Association of Parental and Contextual Stressors With Child Screen Exposure and Child Screen Exposure Combined With Feeding. JAMA Netw Open. 2020 Feb 5;3(2):e1920557. doi: 10.1001/jamanetworkopen.2019.20557. PMID: 32022883.
11. Sundqvist A, Koch FS, Birberg Thornberg U, Barr R, Heimann M. Growing Up in a Digital World - Digital Media and the Association With the Child's Language Development at Two Years of Age. Front Psychol. 2021 Mar 18;12:569920. doi: 10.3389/fpsyg.2021.569920. PMID: 33815187; PMCID: PMC8015860.
12. Taylor G , Monaghan P , Westermann G .Investigating the association between children's screen media exposure and vocabulary size in the UK.Routledge, 2018(1).DOI:10.1080/17482798.2017.1365737.
13. Çelen Yoldaş T, Özmert EN. Communicative Environmental Factors Including Maternal Depression and Media Usage Patterns on Early Language Development. Matern Child Health J. 2021 Jun;25(6):900-908. doi: 10.1007/s10995-021-03125-3. Epub 2021 Apr 27. PMID: 33905063.
14. Kabali HK, Irigoyen MM, Nunez-Davis R, Budacki JG, Mohanty SH, Leister KP, Bonner RL Jr. Exposure and Use of Mobile Media Devices by Young Children. Pediatrics. 2015 Dec;136(6):1044-50. doi: 10.1542/peds.2015-2151. Epub 2015 Nov 2. PMID: 26527548.
15. Shah PE, Hirsh-Pasek K, Kashdan TB, Harrison K, Rosenblum K, Weeks HM, Singh P, Kaciroti N. Daily television exposure, parent conversation during shared television viewing and socioeconomic status: Associations with curiosity at kindergarten. PLoS One. 2021 Oct 28;16(10):e0258572. doi: 10.1371/journal.pone.0258572
16. Çelen Yoldaş T, Özmert EN. Communicative Environmental Factors Including Maternal Depression and Media Usage Patterns on Early Language Development. Matern Child Health J. 2021 Jun;25(6):900-908. doi: 10.1007/s10995-021-03125-3. Epub 2021 Apr 27. PMID: 33905063.
17. Shah PE, Hirsh-Pasek K, Kashdan TB, Harrison K, Rosenblum K, Weeks HM, Singh P, Kaciroti N. Daily television exposure, parent conversation during shared television viewing and socioeconomic status: Associations with curiosity at kindergarten. PLoS One. 2021 Oct 28;16(10):e0258572. doi: 10.1371/journal.pone.0258572
18. Lin Y, Zhang X, Huang Y, Jia Z, Chen J, Hou W, Zhao L, Wang G, Zhu J. Relationships between screen viewing and sleep quality for infants and toddlers in China: A cross-sectional study. Front Pediatr. 2022 Oct 10;10:987523. doi: 10.3389/fped.2022.987523. PMID: 36299700; PMCID: PMC9589267.
19. Zhou SS, Yan SQ, Cao H et al. Prevalence and the risk factors of television viewing by infants and toddlers in Ma′anshan city. Chin J Child Health Care. 2020 Sep;28(1):61-64. doi:10.11852/zgetbjzz2019-1087
20. Zimmerman FJ, Christakis DA, Meltzoff AN. Associations between media viewing and language development in children under age 2 years. J Pediatr. 2007 Oct;151(4):364-8. doi: 10.1016/j.jpeds.2007.04.071. Epub 2007 Aug 7. PMID: 17889070.
21. Shah PE, Hirsh-Pasek K, Kashdan TB, Harrison K, Rosenblum K, Weeks HM, Singh P, Kaciroti N. Daily television exposure, parent conversation during shared television viewing and socioeconomic status: Associations with curiosity at kindergarten. PLoS One. 2021 Oct 28;16(10):e0258572. doi: 10.1371/journal.pone.0258572
22. He Hui, Wei Zhuang, Liu Chunyang, Liang Aimin. Survey on Children's Use of Electronic Products in Beijing. Maternal and Child Health Care of China. January. 2016;31(2):351–353. doi:10. 7620 /zgfybj. j. issn. 1001－4411. 2016. 02. 47
23. FANG Hong-ying, TANG Yin-xia, XU Cheng-heng,YANG Fei-fei,ChEN Shang-hui. Survey on exposure to electronic devices of preschool children in Tongling city. Chinese Journal of Child Health Care, 2018, 26(8): 913-915.
24. WANG Xuena, DU Wenwen, ZHANG Meng, et al. Investigation on screen time and psychosocial problems of preschoolers during the prevalence of COVID—19. China Journal of Health Psychology, 2021;29(4):564–568.
25. SONG Wenya, PAN Xiaowei, SUN Longxia, NI Yu. Effects of screen exposure on cognitive-social development in preschooler.Chines Journal of Woman and Child Health Reserach, 2021;32(10):1452–1457.
26. Shah PE, Hirsh-Pasek K, Kashdan TB, Harrison K, Rosenblum K, Weeks HM, Singh P, Kaciroti N. Daily television exposure, parent conversation during shared television viewing and socioeconomic status: Associations with curiosity at kindergarten. PLoS One. 2021 Oct 28;16(10):e0258572. doi: 10.1371/journal.pone.0258572
27. Taylor G, Monaghan P, Westermann G. Investigating the association between children’s screen media exposure and vocabulary size in the UK. Journal of Children and Media. 2018 Jan 2;12(1):51–65.
28. Takahashi I, Obara T, Ishikuro M, Murakami K, Ueno F, Noda A, Onuma T, Shinoda G, Nishimura T, Tsuchiya KJ, Kuriyama S. Screen Time at Age 1 Year and Communication and Problem-Solving Developmental Delay at 2 and 4 Years. JAMA Pediatr. 2023 Oct 1;177(10):1039-1046. doi: 10.1001/jamapediatrics.2023.3057.
29. 29.	Wu XY, Tao SM, Zhang SC, Zhang YK, Huang K, Tao FB. [Analysis on risk factors of screen time among Chinese primary and middle school students in 12 provinces]. Zhonghua Yu Fang Yi Xue Za Zhi. 2016 Jun;50(6):508-13. Chinese. doi: 10.3760/cma.j.issn.0253-9624.2016.06.007. PMID: 27256730.
30. Tombeau Cost K, Korczak D, Charach A, Birken C, Maguire JL, Parkin PC, Szatmari P. Association of Parental and Contextual Stressors With Child Screen Exposure and Child Screen Exposure Combined With Feeding. JAMA Netw Open. 2020 Feb 5;3(2):e1920557.
31. WANG Wei,WEI Yanan. A Survey Study on Parents' Attitudes toward Young Children's Use of Electronic Products--Taking Chongqing Municipality as an Example. Education Guide (the second half of the month),2017(2):75-78.
32. Li, C. , Cheng, G. , Sha, T. , Cheng, W. , & Yan, Y. . (2020). The relationships between screen use and health indicators among infants, toddlers, and preschoolers: a meta-analysis and systematic review. International Journal of Environmental Research and Public Health, 17(19), 7324.
33. Zhou SS, Yan SQ, Cao H et al. Prevalence and the risk factors of television viewing by infants and toddlers in Ma′anshan city. Chin J Child Health Care. 2020 Sep;28(1):61-64. doi:10.11852/zgetbjzz2019-1087
34. SHAN Ruijie, HAN Jing, QU Keli, YUE Lei, CUI Naixue. Associated factors of screen exposure in infants. Chinese Journal of Child Health Care, 2023, 31(4): 374-378.
